# Balancing innovation and safety in percutaneous treatment of coronary artery calcification

**DOI:** 10.3389/fcvm.2026.1964987

**Published:** 2026-10-27

**Authors:** Ayman Helal, Andrew Ladwiniec, Abdul Mozid, Iqbal Malik, Neil Ruparelia, Mohsin Farooq, F. Aaysha Cader, Javed Ehtisham, Prashanth Raju, Naeem Shaukat, Bhavik Shah, Girish Viswanathan, Bernard Prendergast

**Affiliations:** 1 Department of Cardiology, University Hospitals of Northamptonshire, Kettering General Hospital, Kettering, Northamptonshire, United Kingdom; 2 Department of Cardiology, University Hospitals of Leicester NHS Trust, Leicester, United Kingdom; 3 Yorkshire Heart Centre, Teaching Hospitals NHS Trust, University of Leeds, Leeds, United Kingdom; 4 Department of Cardiology, Hammersmith Hospital, Imperial College Healthcare NHS Trust, London, United Kingdom; 5 Department of Cardiology, Derriford Hospital, University Hospitals Plymouth, Plymouth, United Kingdom; 6 Department of Cardiology, Cleveland Clinic, London, United Kingdom

**Keywords:** balancing innovation, calcium modification, chip, coronary artery calcification (CAC), percutaneous coronary intervention (PCI)

## Abstract

Coronary artery calcification (CAC) is an independent predictor of adverse outcomes in percutaneous coronary intervention (PCI). Under-preparation of calcified plaque predisposes to stent under-expansion, mal-apposition, restenosis, and stent thrombosis, while over-aggressive debulking strategies carry risk of dissection, perforation, and no-reflow. The interventional armamentarium for calcium modification has expanded from high-pressure and non-high pressure specialty balloons including intravascular lithotripsy (IVL) to dedicated atherectomy devices i.e., rotational atherectomy (RA), orbital atherectomy (OA), and excimer laser coronary atherectomy (ELCA), each with a distinct mechanism, evidence base, and risk profile. Additionally, comprehensive assessment of coronary calcium is central to guiding lesion preparation and selecting treatment strategy, with IVUS and OCT providing detailed intravascular characterisation, and CT emerging as a complementary non-invasive tool for procedural planning. Appropriate procedural planning and judicious selection of calcium-modification tools are paramount to maximising procedural safety, minimising complications, and improving clinical outcomes. This review summarizes the pathophysiology of CAC, describes each modification technique, synthesizes the pivotal and recent randomised evidence, outlines current guideline position, and proposes a practical framework for balancing procedural innovation against patient safety. Current evidence supports an individualized, imaging-guided approach to calcium modification. Balloon-based strategies, IVL, atherectomy, and ELCA each have specific strengths and limitations, and no modality has demonstrated universal superiority across lesion subsets. Atherectomy and laser remain important for selected uncrossable, undilatable, or highly resistant lesions, whereas IVL and specialty balloons may provide effective alternatives in appropriately selected crossable lesions. Evidence for many technologies is stronger for procedural and imaging endpoints than for long-term clinical outcomes.

## 1 Introduction

Coronary artery calcification (CAC) is increasingly common as populations age with chronic kidney disease and risk factors for atherosclerotic cardiovascular disease, including diabetes mellitus, smoking, dyslipidaemia, and hypertension. Severe CAC poses difficulty in balloon and stent delivery, prevents full stent expansion, and is an independent predictor of target lesion failure, stent thrombosis, and restenosis (1).

Historically, operators relied on high-pressure balloon dilation alone, a strategy frequently associated with unsatisfactory results in heavily calcified lesions. The subsequent introduction of dedicated calcium-modifying devices has led to improved lesion compliance and stent expansion, but each technology introduces procedural complexity, cost, and device-specific complications (2). Particularly in an already high-risk patient subset, caution is advised in the pursuit of perfection with calcium modification. The central theme explored in this review is that more aggressive calcium modification does not automatically translate into better clinical outcomes, as several contemporary randomised trials have shown.

The principal contribution of this review is to integrate contemporary randomized evidence with an imaging-guided, lesion-specific decision framework for calcified PCI. Rather than ranking calcium-modification devices as universally superior or inferior, we examine how recent trials have changed the threshold for device escalation and emphasize the distinction between procedural success, imaging endpoints, periprocedural safety, and long-term clinical benefit. This framework places innovation within a safety-conscious strategy aimed at achieving sufficient, rather than maximal, calcium modification.

## 2 Methodology

A structured narrative literature search was undertaken to identify evidence relating to coronary artery calcification, intravascular imaging, CT-based coronary calcium assessment, calcium-modification technologies, and outcomes following PCI. MEDLINE/PubMed, Embase, the Cochrane Library, and Google Scholar were searched from database inception to the date of the final search. ClinicalTrials.gov, conference proceedings from major interventional cardiology meetings, guideline documents, and reference lists of relevant publications were also reviewed.

Search terms included combinations of “coronary artery calcification,” “calcified coronary lesion,” “calcium modification,” “rotational atherectomy,” “orbital atherectomy,” “excimer laser coronary atherectomy,” “intravascular lithotripsy,” “cutting balloon,” “scoring balloon,” “intravascular ultrasound,” “optical coherence tomography,” “coronary CT angiography,” “stent underexpansion,” “minimal stent area,” “target-lesion failure,” and “percutaneous coronary intervention.”

Priority was given to contemporary guidelines, randomized controlled trials, prospective comparative studies, large registries, systematic reviews, and mechanistic imaging studies. Single-arm studies, case series, and case reports were included when they addressed uncommon lesion subsets, novel technologies, or bail-out and combination strategies for which comparative randomized evidence was unavailable. Conference-reported studies were included to reflect current developments but were clearly identified as preliminary evidence.

Studies were selected by consensus among the authors according to clinical relevance, methodological quality, directness of evidence, contemporary procedural practice, and relevance to the review's central question of how calcium-modification strategies should be selected while balancing procedural efficacy and safety. This was a narrative review and no formal quantitative synthesis or risk-of-bias meta-analysis was performed.

## 2 Pathophysiology and clinical significance of CAC

CAC develops through cell-mediated processes resembling osteogenesis, with calcium phosphate hydroxyapatite deposition occurring in both the intima and media—especially in elderly patients with diabetes mellitus and/or chronic kidney disease. Calcified plaques subsequently increase arterial stiffness, disrupt normal balloon-artery interaction, and create an irregular luminal surface that can interferes with uniform stent expansion. Superficial, circumferential (≥270°), and long-segment calcification are the morphologic patterns most strongly associated with stent under-expansion and lead to short- and long-term adverse events. Calcified nodules, a distinct entity in which calcium fragments protrude into the lumen, represent a particularly high-risk subset that require different management from circumferential calcific plaque (3).

Coronary calcium is an important marker of plaque burden and provides valuable information about lesion rigidity, fracture resistance, and the likelihood of stent under-expansion. However, structural calcium burden should not be equated with biological plaque activity. Plaque inflammation, macrophage activation, lipid-rich necrotic core, neovascularization, endothelial dysfunction, and thrombogenic potential may influence clinical risk independently of the amount or distribution of calcium visible on angiography, CT, IVUS, or OCT. Thus, anatomical imaging is essential for procedural planning but provides an incomplete assessment of plaque vulnerability and cardiovascular risk. In selected settings, molecular or hybrid imaging may offer complementary information regarding biological activity, although these approaches are not yet established as routine tools for guiding calcium-modification strategy.

## 3 The role of computed tomography coronary angiography (CTCA)

Computed tomography coronary angiography (CTCA) has emerged as a valuable non-invasive modality for pre-procedural assessment of CAC, and provides detailed characterization of calcium burden, calcium score, distribution, arc, thickness, length, and the presence of calcified nodules, and assessment of calcium density and resistance to fracture. Furthermore, CTCA can differentiate between deep, superficial calcium, concentric, and eccentric calcification, and define lesion morphology, allowing operators to tailor their calcium modification technique (4). Pre-procedural CT assessment enables better upfront procedural planning prior to the patient arriving in the cath lab, and facilitates selection of the most appropriate calcium modification strategy.

Sekimoto et al. demonstrated that lesion-specific calcium burden and a calcium arc ≥270° on CT could predict the need for rotational atherectomy (5). More recently, Yang et al. demonstrated that a lesion-specific CAC score >200 AU was independently associated with stent under expansion, supporting a potential role for CT in identifying lesions requiring more intensive calcium modification before stent implantation (6). As CT technology and artificial intelligence continue to evolve, pre-procedural CTCA is increasingly being integrated into precision PCI planning, which will subsequently lead to optimal selection of lesion preparation strategy. The randomised OPTIMAL trial (A Prospective, Multi-centre, Randomised Controlled Trial to Investigate the Value of Coronary CT Angiography in the Understanding and Management of Coronary Calcium; NCT07286578) will evaluate whether pre-procedural CCTA-based characterization of coronary calcium can guide calcium-modification strategy and improve PCI efficiency and stent expansion, compared with an IVUS-guided approach. The study may help define an emerging role for CCTA as a non-invasive tool for selecting calcium-modification techniques and planning complex calcified PCI (7).

The prospective multi-centre P3 study demonstrated that an FFRCT-based virtual PCI planner accurately predicted post-PCI physiological outcomes, with excellent agreement between predicted and measured post-PCI FFR (0.86 vs. 0.88) and consistent accuracy regardless of calcium burden or lesion pattern. These findings support the use of pre-procedural CTCA and FFRCT for procedural planning (8).

## 4 Role of intravascular imaging in assessment of calcium burden

Two-dimensional angiography underestimates the true extent of CAC, demonstrating the lumen silhouette alone and often overlooking calcium that does not distort the vessel contour (9). Intravascular imaging including intravascular ultrasound (IVUS) and optical coherence tomography (OCT), has therefore become crucial in dealing with CAC at three distinct stages of the procedure: pre-procedural lesion characterization, intra-procedural device selection and assessment of the effectiveness/ adequacy of calcium modification, and post-procedural confirmation of adequate stent expansion (10).

### 4.1 Pre-procedural lesion characterization

OCT can quantify the three variables most predictive of stent under-expansion: 1) calcium arc (circumferential extent, with ≥270° considered severe), 2) calcium thickness, and 3) calcium length (11).

IVUS, has a tissue penetration depth of up to 10 mm, and is well suited to the assessment of superficial and deep calcium, external elastic lamina (EEL) dimensions and overall plaque burden. Calcium is typically demonstrated as an echo-bright leading arc signal with acoustic shadowing. However, true CAC thickness cannot be determined since ultrasound is reflected and does not penetrate through calcium (12). Zhang et al. proposed a 4-point IVUS-based pre-PCI scoring system wherein a total score of greater than or equal to 2 was predictive of stent under expansion (13).

OCT has remarkably higher axial resolution (∼10–15 μm vs. ∼100–150 μm for IVUS) but shallower tissue penetration (∼1–3 mm vs. ∼10 mm for IVUS), enabling direct measurement of calcium arc, thickness, and length with much greater precision. A validated 4-point pre-procedural OCT calcium score by Fujino et al. predicted stent under expansion in calcified lesions (14). OCT is particularly well suited to identifying calcified nodules (that are difficult to distinguish from surrounding plaque on IVUS or angiography alone) (15).

This distinction matters clinically: many operators prefer to use IVUS as a first-imaging tool to size vessels and assess overall plaque burden, reserving OCT for the detailed evaluation of calcium morphology and depth, and nodule detection when a nodule-specific strategy is planned (16).

### 4.2 Intra-procedural device selection

Imaging findings translate directly into appropriate device choice. Superficial, thick, and long calcific segments with a wide arc generally favour a dedicated debulking strategy (rotational or orbital atherectomy, or IVL) over balloon-based lesion preparation, whereas more modest or eccentric calcium may be adequately treated with scoring or cutting balloons alone (17). A Zhang IVUS score of ≥2 indicates a high risk of poor stent expansion and suggests the need for up-front plaque modification (such as atherectomy) (13). Calcified nodules are a special form of CAC and identified as a calcium mass with a convex structure that protrudes into the vessel lumen. They are usually more resistant and difficult to manage with standard single calcium modification modality and usually approached with sequential or combination strategies (e.g., atherectomy followed by IVL) (18).

### 4.3 Post-procedural outcome

Intravascular imaging is used to confirm that stent apposition and expansion are adequate—an endpoint that is directly linked to downstream outcomes. In the OCT sub-study of the ECLIPSE trial, there was no statistically significant difference in minimal stent area (MSA) between the orbital atherectomy and balloon angioplasty arms. However, secondary analysis found that IVUS/OCT- guided PCI was associated with lower rate of target vessel failure than angiographic guidance PCI at 1-year (9.3% vs. 13.2%). Surprisingly, this benefit was independent of lesion-preparation strategy (orbital atherectomy or balloon angioplasty), reinforcing the fact that the choice of imaging guidance may matter as much as -or more than- the mode of calcium modification (19).

## 5 Calcium modification techniques

### 5.1 Conventional and specialty balloons

Standard non-compliant balloons remain the default first step for most calcified lesions but frequently fail to achieve adequate expansion when calcium is severe, because force is distributed longitudinally and circumferentially rather than focused on the calcific plate (20).

Specialty balloons attempt to concentrate dilating force in specific areas to facilitate controlled cutting of calcific lesions:

- Cutting balloons are loaded with three or four microtomes (blades) bonded longitudinally to the balloon surface. which score the calcific plaque to create controlled fracture points and reduce balloon slippage (watermelon-seeding) during slow inflation (21).
- Scoring balloons are specialized balloons wrapped with external wire elements (such as nitinol struts) and achieve a similar focal-force effect with lower profile, that is often useful for calcified lesions in smaller or more distal vessels (22).
- Ultra-high-pressure non-compliant balloons (to 35–40 ATM) provide an alternative focal-force without cutting or scoring elements and are useful particularly for resistant, non-expandable lesions after preparation with conventional non-compliant balloons (23).

Conventional and specialty balloons are less expensive, technically straightforward to use, and widely available. On the other hand, their effectiveness drops substantially in severe, circumferential, or deep calcification, as they generate insufficient force to fracture the calcific plate with increased risk of balloon rupture or vessel injury and inadequate lesion modification. Despite this theoretical ceiling, recent randomised data suggest that specialty balloons perform better against dedicated debulking technologies than previously assumed. The Short-Cut trial, presented at TCT 2025, randomised 413 patients with moderate-to-severely calcified *de novo* coronary lesions across 21 U.S. sites to cutting balloon vs. IVL before drug-eluting stent implantation. Cutting balloon was noninferior to IVL for the primary endpoint of post-procedural minimal stent area (MSA) at the site of maximum calcification (8.0 mm^2^ vs. 8.6 mm^2^), with no significant differences between groups in stent expansion, calcium fracture rates, procedural success, intraprocedural adverse events, or 30-day MACE. Notably, the cutting-balloon strategy was also substantially less expensive, saving approximately $3,600 per case, driven primarily by device cost rather than other procedural factors. Similarly, the parallel VICTORY trial presented at the same meeting found the OPN ultra-high-pressure non-compliant balloon to be noninferior to IVL for OCT-measured final stent expansion, with comparable safety and early clinical outcomes. Together, these findings indicate that specialty balloons can achieve comparable results to costlier dedicated calcium-modification devices in appropriately selected, imaging-confirmed lesions (2).

Recently presented studies, including Short-CUT and VICTORY, suggest that selected specialty-balloon strategies may be non-inferior to IVL for procedural imaging endpoints such as minimal stent area or final stent expansion. These findings are clinically important but should not be interpreted as proof of equivalent long-term clinical safety or efficacy. The available results are primarily based on mechanistic endpoints and, at the time of writing, do not provide the same evidentiary weight as mature peer-reviewed randomized trials powered for clinical outcomes. Their applicability is also dependent on lesion selection and operator expertise.

### 5.2 Rotational atherectomy (RA)

RA is the oldest dedicated calcium-modification technology and uses a diamond-coated, elliptical burr rotating at high speed (typically 135,000–180,000 rpm) over a dedicated guidewire, ablating inelastic calcified tissue by differential cutting while sparing more elastic, healthy arterial tissue. Limitations include guidewire bias (the burr tends to ablate along the path of least resistance rather than uniformly), the risk of slow flow/no-reflow resulting from embolization of microparticulate debris, burr entrapment, coronary dissection or perforation, and a demanding learning curve (24).

Key randomised evidence for RA:

- The ROTAXUS trial (2013) compared RA followed by paclitaxel-eluting stent implantation with a balloon-only strategy before stenting in complex calcified lesions. RA improved acute lumen gain and reduced strategy crossover but did not improve 9-month angiographic and clinical outcomes, or late lumen loss (25).
- The PREPARE-CALC trial (2018) compared upfront RA with a modified balloon (cutting/scoring) strategy before drug-eluting stent implantation and showed similar late-loss and MACE outcomes with both strategies, RA being associated with higher procedural success but more crossover in the balloon arm (26).
- Pooled analysis of ROTAXUS and PREPARE-CALC trials (440 patients) found significantly more crossover from the balloon-only control arm to atherectomy (14.1% vs. 2.3%), resulting in higher procedural success in the RA group, and no difference in late lumen loss or MACE at 9 months (27).

### 5.3 Orbital atherectomy (OA, “diamondback 360”)

OA uses an eccentrically mounted, diamond-coated crown that orbits within the vessel (rather than rotates concentrically), ablating calcium in both antegrade and retrograde passes while allowing continuous blood flow around the crown (which may reduce ischaemia and heat generation compared with RA). Early single-arm evidence (ORBIT I and ORBIT II studies) supported feasibility and reasonable safety, leading to widespread adoption (28).

The ECLIPSE trial was a pivotal study that randomised 2,005 patients at 104 U.S. centres to OA vs. conventional balloon angioplasty before second-generation drug-eluting stent implantation. The primary clinical endpoint of target vessel failure at one year occurred in 11.5% of the OA group compared with 10.0% of the balloon angioplasty group, and the primary imaging endpoint (mean post-PCI MSA at the site of maximum calcification) was 7.67 mm^2^ with OA vs. 7.42 mm^2^ with conventional PCI—neither difference reaching significance. Coronary perforation occurred in 1.8% of the orbital atherectomy group vs. 1.0% of the conventional PCI group, although cardiac deaths were significantly higher after OA at 30 days (0.8% vs. 0.0%), several being device related. The trial concluded that routine OA did not improve stent expansion or reduce target vessel failure compared with a simpler balloon-based strategy, and that use of OA should not be applied routinely but individualized based upon lesion and patient characteristics. This recommendation directly challenges the long-standing assumption that routine debulking of severe calcium improves outcomes and is arguably the single most important recent data point reshaping practice in this field (29). A pre-specified OCT ECLIPSE sub-study similarly found no significant difference in MSA between OA and balloon angioplasty arms, reinforcing the neutral primary result at mechanistic level (19).

The neutral ECLIPSE findings apply primarily to severely calcified lesions that were considered suitable for both orbital atherectomy and conventional balloon angioplasty. They should not be generalized to uncrossable or undilatable lesions, lesions requiring atherectomy to facilitate device passage, or highly selected nodular lesions in which a different or sequential strategy may be necessary.

### 5.4 Coronary intravascular lithotripsy (IVL, “shockwave”)

IVL uses lithotripsy emitters integrated into a conventional semi-compliant balloon catheter to deliver localized, pulsatile acoustic (sonic) pressure waves through the vessel wall circumference -selectively fracturing calcium while sparing surrounding soft tissue- followed by low-pressure balloon inflation to achieve luminal gain. The technique is compatible with standard PCI workflow and does not require a specialized atherectomy guidewire and is generally regarded as less operator-dependent than rotational or orbital atherectomy (30).

The serial Disrupt CAD I–IV trials, pooled in several analyses, form the core evidence base. Across 628 patients enrolled at 72 sites in 12 countries, severe calcification was confirmed in 97.0% of target lesions, and the primary safety and effectiveness endpoints were achieved in 92.7% and 92.4% of patients, respectively. At 30 days, rates of target lesion failure, cardiac death, and stent thrombosis were 7.2%, 0.5%, and 0.8%, with serious angiographic complications occurring in only 2.1% (post-IVL) and 0.3% (final), and no IVL-associated perforations, abrupt closure, or no-reflow. A dedicated Japanese cohort within Disrupt CAD IV extended these findings to an Asian population with durable 1-year safety and effectiveness. A pooled OCT analysis focused specifically on calcified nodules found that although these lesions carried a higher baseline calcium burden, IVL achieved comparable stent expansion and 2-year target lesion failure rates compared with non-nodular calcified lesions, suggesting IVL is a reasonable option even in this historically difficult subset (30).

Head-to-head comparisons with RA have also been reassuring for IVL. The ROTA shock trial randomised 70 patients to IVL or RA before PCI of severely calcified lesions, with OCT-assessed MSA (the primary endpoint) being numerically lower (but statistically non-inferior) after IVL (6.10 mm^2^) compared with RA (6.60 mm^2^), and stent expansion remaining similar in both groups (31). Data from the recently published ICARE trial (see below) support these observations, while the three-arm ROLLER COASTR-EPIC22 trial (see below) extended this comparison to include laser atherectomy.

With regards to these trials, it is to be noted that non-inferiority is endpoint-specific and depends on the prespecified non-inferiority margin, analysis population, event rate, and trial power.

### 5.5 Excimer laser coronary atherectomy (ELCA)

ELCA delivers pulsed ultraviolet (308 nm) energy through flexible fibre-optic catheters, ablating tissue through a combination of photochemical, photothermal, and photomechanical effects. Unlike RA and OA, ELCA does not require a dedicated stiff guidewire, and is therefore particularly useful for calcified lesions that are difficult to cross with a microcatheter or balloon, as well as for in-stent restenosis, chronic total occlusions (CTO), and for degenerative saphenous vein grafts, in addition to calcified lesions. Modern smaller-diameter (0.9 mm) catheters have improved the safety profile compared with older-generation devices, whilst technical refinements such as saline/contrast flushing during laser application (MAXCon ELCA) enhance ablation efficiency and reduce the risk of dissection or perforation. Historically, evidence supporting the specific use of ELCA for modification of calcified plaque has been comparatively limited, consisting of registries and case series rather than large, randomised trials. As a consequence, ELCA has generally been considered a second-line or adjunctive tool for use when primary calcium-modification techniques fail to cross the lesion (32).

### 5.6 Head-to-head comparisons and combination strategies

Clinically, the most important recent development has been reporting of direct, randomised, three-way comparisons of the dedicated debulking technologies:

- ROLLER COASTR-EPIC22 was the first randomised trial to directly compare RA, IVL, and ELCA in the same population and enrolled 171 patients with moderate-to-severe calcified coronary lesions (57 per arm) across 8 high-volume Spanish centres, with OCT-assessed stent expansion as the primary noninferiority endpoint. Severe angiographic calcification was present in 82.5% of lesions, and procedural success and final MSA similar in the three arms (RA 5.5 mm^2^, IVL 5.4 mm^2^, ELCA 5.1 mm^2^). Furthermore, there was no significant differences in the composite MACE endpoint at one-year follow-up. IVL was non-inferior to RA in stent expansion, while ELCA did not meet this threshold. No significant differences were found between the three treatments in terms of MSA, procedural success, or complications, although complications were numerically lower with IVL (33) A *post-hoc* comparison with the smaller ROTA shock trial noted broadly similar stent expansion despite more severe calcification in ROLLER COASTR-EPIC22, reinforcing the notion that RA, IVL, and ELCA are complementary rather than hierarchically superior options—no single technology is adequate for every lesion morphology (31, 33).
- RotaTripsy/ELCA-tripsy—combining RA or laser with subsequent IVL—has emerged as a bail-out or planned strategy for very resistant or nodular calcium that fails with a single-modality approach, supported mainly by registries (e.g., the Rota-Shock registry) and case series rather than randomised data (33–36).
- Laser intravascular lithotripsy (L-IVL) is a newer hybrid concept that combines the lesion-crossing advantages of laser with lithotripsy-style calcium fracture. *Ex vivo* testing has confirmed that L-IVL effectively increases the compliance and lumen area of heavily calcified human coronary arteries by disrupting plaque through multiple mechanical actions resulting in significant increase in lumen area and enhanced arterial compliance. Advanced imaging and simulations confirm that this success is driven not only by visible plaque fractures (in 78% of samples) and hidden microscopic cracks (in 57% of samples) but also by tissue debonding and intimal tear (36).
- The ICARE OFDI randomised trial compared IVL with RA in 169 patients undergoing OCT guided PCI for moderate to severe CAC and concluded that IVL was non-inferior to RA in achieving the primary endpoint of MSA (6.0 ± 2.3 mm^2^ vs. 5.9 ± 2.2 mm^2^). Both strategies produced similar rates of adequate stent expansion (RA: 65.1%, IVL: 65.1%; *p* = 0.99) with comparable procedural safety and low rates of target lesion failure at 1 year, although the incidence of major stent strut mal-apposition was significantly less after IVL than RA. These findings support IVL as an effective and safe alternative to RA for the treatment of heavily calcified coronary lesions (37).

It is to be noted that numerical differences in adverse events should be interpreted cautiously when the study is not powered to detect differences in clinical safety outcomes. A statistically non-significant result indicates that a difference was not demonstrated; it does not establish equivalence or prove that one device is safer than another.

## 6 Comparative summary

Tables 1, 2 summarises the principal differences between calcium modification techniques.

**Table 1:** Differences between calcium modification techniques.

Technique	Mechanism	Key advantages	Key limitations	Pivotal/recent trials
Cutting/scoring balloon	Focal mechanical force	Simple, low cost	Ineffective in severe/circumferential calcium	Adjunctive use mainly; comparator arm in PREPARE-CALC
Rotational atherectomy	High-speed differential ablation	Effective in un-dilatable/fibrotic calcium	Guidewire bias, no-reflow, learning curve	ROTAXUS, PREPARE-CALC, ROTA.shock, ROLLER COASTR-EPIC22
Orbital atherectomy	Eccentric orbiting ablation, antegrade/retrograde	Continuous flow around crown	No outcome benefit over balloon in RCT; perforation/mortality signal	ORBIT I/II, ECLIPSE
Intravascular lithotripsy	Acoustic pressure wave calcium fracture	Low operator dependence, favourable safety profile	Requires lesion crossing with standard balloon first	Disrupt CAD I–IV, ROTA.shock, ROLLER COASTR-EPIC22
Excimer laser atherectomy	Photochemical/thermal/mechanical ablation	Crosses uncrossable lesions without dedicated wire	Limited randomised calcium-specific data	Registries; ROLLER COASTR-EPIC22

**Table 2:** Summative comparison of randomised controlled trials comparing different calcium modification techniques. .

Study	Design and population	Primary endpoint	Main result	Clinical outcomes	Key limitation
ROTAXUS	Randomized comparison of RA versus balloon-based preparation	Angiographic and clinical outcomes	Improved acute lumen gain and reduced crossover with RA; no clear late clinical benefit	No significant improvement in late outcomes	Older stent technology and limited sample size
PREPARE-CALC	Randomized comparison of RA versus modified balloon strategy	Procedural success and late angiographic outcomes	Greater procedural success with RA; similar late-loss and MACE outcomes	No clear clinical superiority	Crossover and modest power
ECLIPSE	Large randomized comparison of OA versus conventional balloon angioplasty in lesions suitable for both strategies	Target-vessel failure and minimal stent area	No significant improvement with routine OA	Neutral one-year target-vessel failure result; device-specific safety signals	Not applicable to uncrossable or undilatable lesions
ROTA.shock	Small randomized comparison of RA versus IVL	OCT-derived minimal stent area	IVL non-inferior for the prespecified imaging endpoint	Underpowered for clinical events	Small sample and imaging-focused endpoint
ICARE OFDI	Randomized OCT-guided comparison of IVL versus RA	Minimal stent area and stent expansion	Similar minimal stent area and expansion; less major mal-apposition with IVL	Limited clinical-event power	Small sample and mechanistic primary endpoint
ROLLER COASTR-EPIC22	Three-arm randomized comparison of RA, IVL, and ELCA	OCT-assessed stent expansion	Similar procedural and imaging outcomes; IVL non-inferior to RA for the primary endpoint	No robust differences in one-year MACE	Small sample and limited power for clinical outcomes
Short-CUT	Recently presented randomized comparison of cutting balloon versus IVL	Minimal stent area	Cutting balloon non-inferior for the reported imaging endpoint	Early clinical outcomes only	Conference-level or preliminary evidence and imaging-focused endpoint
VICTORY	Recently presented randomized comparison of ultra-high-pressure balloon versus IVL	Final stent expansion	Reported non-inferiority for the imaging endpoint	Early safety outcomes only	Preliminary evidence and limited long-term clinical follow-up

## 7 Guideline perspectives

The 2021 ACC/AHA/SCAI Guidelines for Coronary Artery Revascularization and 2024 ESC Guidelines for the Management of Chronic Coronary Syndromes both recognise dedicated calcium-modification technologies as reasonable adjuncts to facilitate stent delivery and optimise expansion in severely calcified lesions that are not adequately prepared by conventional balloon angioplasty, both guidelines also favour a “provisional” or lesion-specific approach—selecting a debulking strategy based upon lesion morphology, guidewire crossability, and calcium distribution determined by intravascular imaging—rather than mandate routine debulking of every calcified lesion. They also emphasise that intravascular imaging (IVUS or OCT) should guide patient selection for calcium modification and confirmation of adequate stent expansion (38, 39). The neutral results of ECLIPSE (19) and ROLLER COASTR-EPIC22 (33) are likely to reinforce this individualized, imaging-guided philosophy in future guideline updates rather than a strategy of routine atherectomy or lithotripsy for all severely calcified lesions. Hence, intravascular imaging should be utilised for lesion assessment and optimization where feasible.

It is noteworthy that recommendations for calcium-modification devices are often framed as reasonable or useful procedural strategies rather than as mandates for routine use of a particular device in every calcified lesion.

## 8 Balancing innovation and safety

Several themes emerge that speak directly or central question the incidence of major stent strut mal apposition was significantly less after debulking is not automatically beneficial.

### 8.1 The objective of calcium modification

The appropriate calcium-modification strategy should be determined by the interaction between lesion morphology, procedural feasibility, patient risk, and local expertise. The objective is not maximal calcium removal but sufficient modification to permit device delivery, achieve an adequately expanded and apposed stent, and minimize procedural injury. Both ROTAXUS/PREPARE-CALC, and ICARE (for RA) and ECLIPSE (for OA) failed to show superiority of routine atherectomy over simpler balloon-based strategies with respect to late angiographic or one-year clinical outcomes, despite improved acute procedural metrics. This is a critical safety lesson—acute imaging success does not always translate into downstream clinical benefit, and exposure of patients to the added procedural risk of atherectomy should be justified by lesion-specific need (e.g., an un-dilatable or uncrossable lesion) rather than calcium severity alone.

### 8.2 Lesion and imaging features guiding device selection

Device-specific safety signals matter. The numerically higher incidence of perforation and significantly higher cardiac mortality associated with OA use in the ECLIPSE trial provide a reminder that mechanical debulking technologies carry significant device-attributable risk that must be weighed against anticipated benefit, especially in an era where IVL offers comparable lesion preparation with a more favourable safety profile. Lesions that are crossable and adequately dilatable may often be treated with conventional or specialty balloons, particularly when calcium is eccentric, superficial, or of limited longitudinal extent. Lesions with deep, circumferential, or long-segment calcium may require intravascular lithotripsy or atherectomy, depending on whether the lesion can be crossed with the relevant device and whether balloon expansion is possible. Atherectomy or laser may be particularly valuable in uncrossable or undilatable lesions, whereas IVL is generally most useful after successful device crossing when calcium fracture is required without tissue debulking. Calcified nodules may require a sequential or combination strategy, guided by intravascular imaging.

### 8.3 Patient and procedural factors affecting the risk-benefit balance

No single technology is universally superior: ROLLER COASTR-EPIC22 found similar stent expansion and 1-year MACE with RA, IVL, and ELCA. The mechanistic differences of each strategy (guidewire dependence, need for lesion crossability, nodular vs. diffuse calcium) mean that operators benefit from the availability of multiple tools with selection based upon lesion anatomy rather than depending upon a single default technology. The balance between efficacy and safety also depends on the clinical setting. In patients with acute coronary syndromes, haemodynamic instability, severe left ventricular dysfunction, advanced chronic kidney disease, or limited physiological reserve, avoidance of prolonged instrumentation, distal embolization, slow flow, and perforation becomes especially important. Conversely, in an elective patient with a severely calcified, non-dilatable lesion and adequate operator and institutional expertise, the potential benefit of atherectomy or multimodality treatment may justify greater procedural complexity.

### 8.4 Interpretation of procedural, imaging, and clinical endpoints

Combination and bail-out strategies are expanding. RotaTripsy, ELCA-tripsy, and planned trials such as NODULE-SHOCK (https://clinicaltrials.gov/study/NCT07000045) reflect a maturing recognition that very resistant or nodular calcium may require sequential, multimodal preparation, and that innovation is increasingly directed at combined modalities rather than replacing one device with another. Procedural efficacy, imaging success, periprocedural safety, and long-term clinical benefit should be considered separately. Improvement in minimal stent area or calcium fracture demonstrates a mechanistic effect but does not, by itself, establish superiority in target-lesion failure, myocardial infarction, or mortality. The appropriate endpoint is therefore an individualized balance between achieving a clinically meaningful stent result and avoiding unnecessary procedural risk.

### 8. 5 A practical escalation algorithm (Central illustration)

Imaging-guided, individualized practice is the direction of travel. Current U.S. and European guidelines recommend imaging guided patient selection and confirmation of the need for calcium modification rather than routine or empiric device use, applying procedural innovation within a more conservative, safety-conscious framework.

**CENTRAL ILLUSTRATION:**
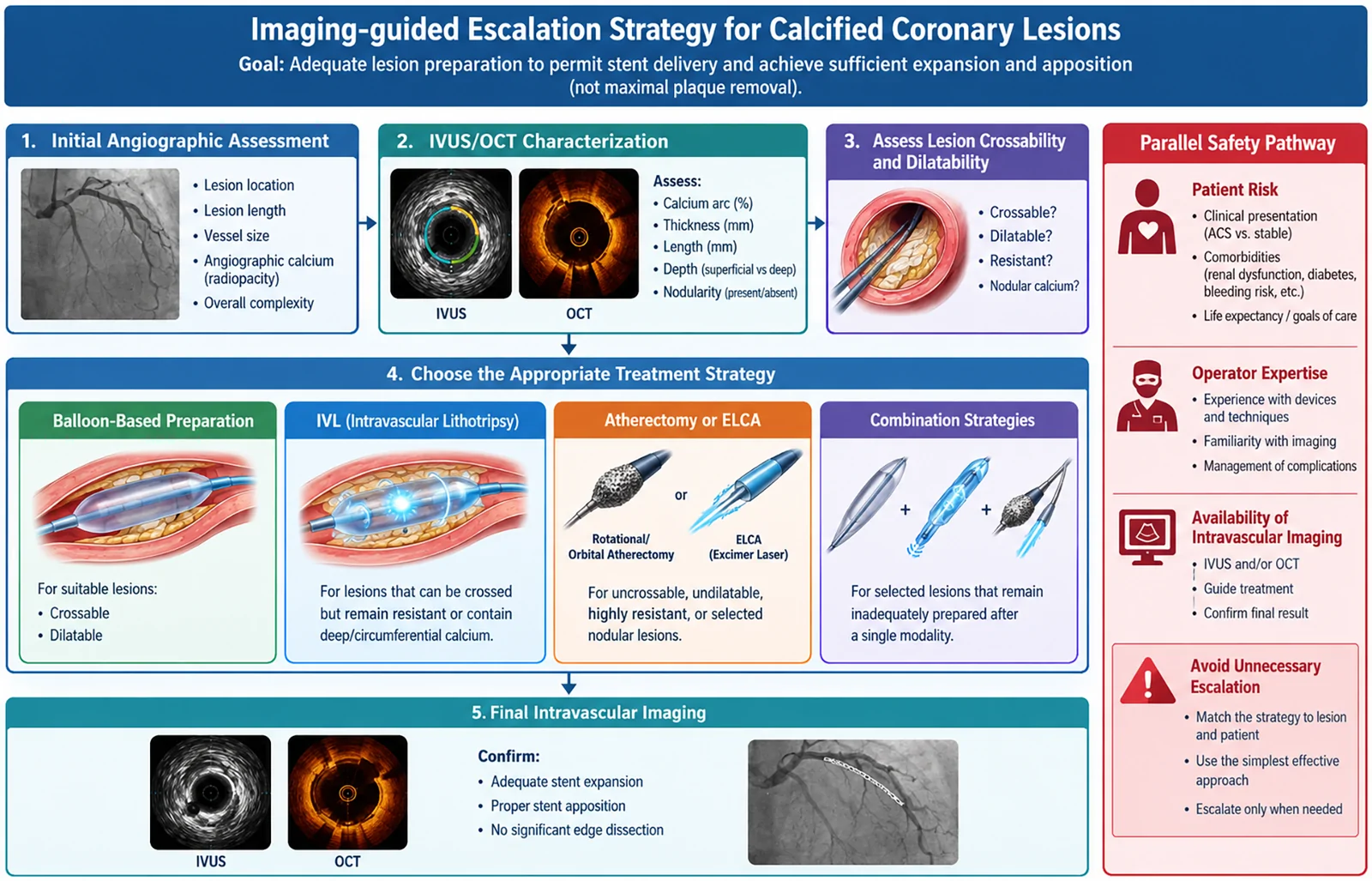
Imaging-guided escalation strategy for calcified coronary lesions. The goal of calcium modification is sufficient lesion preparation to permit stent delivery and achieve adequate expansion and apposition, rather than maximal plaque removal. Lesion morphology, calcium depth and distribution, crossability, dilatability, presence of a calcified nodule, clinical presentation, patient comorbidity, operator expertise, and availability of intravascular imaging should determine device selection. Balloon-based strategies may be appropriate for crossable and dilatable lesions. Intravascular lithotripsy is useful when a balloon can cross but calcium remains resistant, particularly when deep or circumferential calcium is present. Atherectomy or excimer laser may be required for uncrossable, undilatable, or highly resistant lesions. Combination treatment should be reserved for selected lesions that remain inadequately prepared after a single modality. Intravascular imaging should be used whenever feasible to guide treatment and confirm the final result.

## 9 Limitations of the current evidence base

Several caveats deserve emphasis when evaluating the available literature.

- Many pivotal single-arm registries predate contemporary stent technology and imaging-guided practice, limiting direct comparability with newer trials.
- The heterogeneity of calcified lesions and lesion-specific utility of debulking and calcium modification strategies render direct head-to-head comparisons difficult, as clear equipoise for strategies often do not exist for a specific modality.
- Randomised comparisons between individual atherectomy modalities and IVL (e.g., ROTA.shock, ROLLER COASTR-EPIC22) are of modest size (70–171 patients) compared to ECLIPSE and the pooled Disrupt CAD cohort—a better powered for imaging surrogates (MSA, stent expansion) than hard clinical endpoints.
- Most trials are industry-sponsored or industry-supported to varying extent—whilst this, is typical for device research in this space. Comparative claims should be interpreted cautiously.
- Most large trials have enrolled predominantly stable, elective patients—while further information is accumulating, data specific to acute coronary syndromes and calcified nodules remain comparatively limited and these areas are the focus of ongoing dedicated trials.
- A larger MSA or greater stent expansion is mechanistically desirable but does not automatically establish improved long-term clinical outcomes.
- It is also noteworthy that many comparative studies are underpowered for clinical events. Hence, larger randomized controlled trials comparing different modalities for coronary calcium modification with long-term clinical endpoints are of utmost importance and are welcomed.
- The external validity of the available evidence is limited. Most randomized trials enrolled stable, elective patients with lesions considered suitable for the tested strategies and were conducted in experienced centres.
- Patients with acute coronary syndromes, advanced chronic kidney disease, severe left ventricular dysfunction, haemodynamic instability, complex chronic total occlusions, degenerated vein grafts, calcified nodules, very small vessels, and uncrossable or undilatable lesions are comparatively underrepresented. These patients may experience different rates of distal embolization, slow flow, perforation, contrast-associated kidney injury, and haemodynamic compromise. Consequently, trial findings should not be applied uncritically to high-risk anatomical or clinical subgroups.

## 10 Conclusions

The field of calcium modification at the time of PCI has moved from an era in which more aggressive debulking was often assumed to be better, to one increasingly shaped by neutral or cautionary randomised data that argue for individualised, imaging-guided, lesion-specific selection.

Dedicated and expensive atherectomy technologies (including ELCA, RA and OA) retain a well-defined niche for truly un-dilatable or uncrossable lesions with better acute results albeit with increased risk of procedural-related adverse events. In contrast, balloon-based approaches may have practical safety advantages in selected settings because they avoid some atherectomy-specific risks, such as particulate embolization, burr or crown entrapment, and device-related vessel injury. However, balloon techniques may also cause dissection, perforation, balloon rupture, geographic miss, or inadequate lesion preparation. The comparative safety of a device depends on lesion morphology, operator experience, procedural context, and patient risk. IVL has demonstrated a favourable safety profile in available studies, but direct comparisons remain limited and are not uniformly powered for clinical outcomes.

Contemporary calcium modification should be guided by lesion morphology, procedural feasibility, patient risk, and operator expertise rather than by calcium severity alone. The goal is sufficient lesion preparation to achieve an adequately expanded and apposed stent while minimizing unnecessary procedural injury. Atherectomy and ELCA retain important roles in selected uncrossable, undilatable, or highly resistant lesions, whereas IVL and specialty balloons may be appropriate for many crossable lesions with deep, circumferential, or focal calcium. Randomized trials have generally demonstrated improvements in procedural or imaging endpoints more consistently than improvements in long-term clinical outcomes. Accordingly, no technology should be considered universally superior, and non-inferiority for an imaging endpoint should not be interpreted as proof of equivalent long-term safety or efficacy.
